# Data maps and method for evaluating the indicator of the risk of propagation of invasive exotic plant species on work zones

**DOI:** 10.1016/j.dib.2018.06.038

**Published:** 2018-06-26

**Authors:** Samira Mobaied, Elvia Marcellan, Nathalie Machon

**Affiliations:** a Centre d׳Ecologie et des Sciences de la Conservation (CESCO, UMR7204), Muséum national d׳Histoire naturelle, CNRS, Sorbonne Universités, 61 rue Buffon, 75005 Paris, France; b Le CRIGEN Centre de Recherche et d׳expertise Opérationnelle du Groupe ENGIE, 361 Avenue du Président Wilson, 93210 Saint-Denis, France

## Abstract

Invasive exotic plant species are considered a serious threat to plant diversity. Creating work zones involves actions that disturb balances existing among species in the ecosystems and thus promote the propagation and the development of invasive plants. In this context, the Gaz Reseau Distribution France «GRDF», the major french natural gas distributor, with the support of the French National Museum of Natural History «MNHN» and the Research center focused on innovation in gas and new energy sources ENGIE Lab CRIGEN, sought to develop a method for evaluating the risk of propagation of invasive alien plant species in GRDF work zones. When these kind of species are identified, in a woks zone the method calculates the risk of propagation, and depending on the case, specific actions can be recommended. The aim of this paper is to provide data maps and to explain the method developed for the calculation of the risk of propagation of invasive alien plant species in work zones.

**Specifications Table**

**Table:** 

Subject area	Conservation Ecology
More specific subject area	Invasive alien plant, Biodiversity
Type of data	Maps and Table
How data was acquired	Calculating and mapping index
Data format	Maps and Tables
Experimental factors	N/A
Experimental features	N/A
Data source location	France
Data accessibility	Data is provided in the paper

**Value of the data**

• The data presented here could feed a decision-making tool coupled to a GIS (geographic information system); to be used by researchers in order to verify the possible presence of invasive alien plant species in work zones.
• The method developed here enables the calculation of an indicator of the risk of propagation of invasive alien plant species on work zones (IRPW).
• The IRPW indicator classifies work zones likely to promote the propagation of invasive alien plant species based on the spatial context in which the work zones are situated.
• The specific coefficient, presented here, classifies invasive exotic plant species **(IEPS)** according to three factors: (1) The impact on human health of the species, (2) The distance of seed dissemination and (3) regulatory status of the species.

## 1 Data

The data presented here could feed a decision-making tool coupled to a GIS (geographic information system) [17]

1. Map of the number of invasive exotic plant species by town (〈https://qgiscloud.com/Mobaied_S/Data_NbrIPS_Avril_2018/?st=&e=-259108%3B6190783%3B1547996%3B7030835&crs=EPSG%3A2154&t=Data_NbrIPS_Avril_2018&l=Nbr_IPS2018%5B5%5D〉) A map Number of invasive exotic plant species by town that represents the total number of IEPS present in each district was generated by combining the INPN data [15] for the 19 species taken into account by this method. The corresponding table contains the districts identified by their INSEE number [11], and for each district the number and the names of IAPS present.
2. The map labeled (**FOM_IPS_Data_Mobaied**), contains on the corresponding table the towns identified by the INSEE number and 19 columns; each column contains **the frequency of multi-scale observation of the corresponding species**. The name of the column contains that number of the species according to the list shown in Table 1 and the initials of the Latin name of the species. By example, Esp1 is Acer negundo L.

**Table 1:** 19 species identified in the guide to the identification and management of invasive exotic plant species.

**EEVE_NOM latin**	
Esp1	*Acer negundo L.*
Esp2	*Ailanthus altissima (Mill.) Swingle*
Esp3	*Ambrosia artemisiifolia L.*
Esp4	*Baccharis halimifolia L.*
Esp5	*Buddleja davidii Franch.*
Esp6	*Carpobrotus edulis (L.) N.E.BR.*
Esp7	*Cortaderia selloana (Schult. & Schult.f.) Asch. & Graebn.*
Esp8	*Heracleum mantegazzianum Sommier & Levier*
Esp9	*Impatiens glandulifera Royle*
Esp10	*Phytolacca americana L.*
Esp11	*Reynoutria japonica Houtt.*
Esp12	*Reynoutria sachalinensis (F.Schmidt) Nakai*
Esp13	*Rhus typhina L.*
Esp14	*Robinia pseudoacacia L.*
Esp15	*Senecio inaequidens DC.*
Esp16	*Solidago canadensis L.*
Esp17	*Solidago gigantea Aiton*
Esp18	*Ludwigia grandiflora Zardini, H.Y.Gu & P.H.Raven*
Esp19	*Ludwigia peploides Kunth) P.H.Raven*
3. For each of the 19 species identified in the guide, a **map of favorable habitats** was created based on Corine Land Cover by selecting only the land use categories that correspond to the habitats likely to be colonized by the species. These habitats were identified for each species by searching on the descriptive sheets of invasive alien plant species 2., 3., 4., 5., 6., 12.
  In the map labeled (**CLC12_IPS_Data_Mobaied**), the corresponding table contains the CLC categories and the code assigned to each habitat. Nineteen columns were added; the name of the column contains the number of the species according to the list shown in Table 1 and the initials of the Latin name of the species. Each column contains (Yes) when the CLC category corresponds to a favorable habitat for the species mentioned.
4. A map labeled **(PI Map_Data)** depicting all of the highways, rivers, and railways was generated in order to calculate the shortest distance between the work zone and one of these three categories.
5. Tables:

(Table 1): The invasive exotic plants species that were taken into account for this method are the 19 species identified in the Guide to the Identification and Management of Invasive Exotic Plant Species [9].

Table 2: Specific coefficient for each invasive species (1) The health impact of the species coefficient values, (2) The distance of seed dissemination coefficient values (3) Regulation of the species coefficient values and the Specific Coefficient values (the average of the three coefficients for each species).

**Table 2:** Specific coefficient for each invasive species (1) The health impact of the species coefficient values, (2) The distance of seed dissemination coefficient values (3) Regulation of the species coefficient values and the Specific Coefficient values (the average of the three coefficients for each species).

**Species**	**coefficient_health impact of the species**	**Seed dissemination coefficient values**^a^	**Regulated species coefficient values**^b^	**Specific Coefficient**
	No risk for human health: the value of the health impact coefficient is 1	The seed dispersal distance is less than 100 m; the value of the coefficient is 1.	Non-regulated species: the value of the coefficient for these species is 1.	
	Moderate health risk: the value of the health impact coefficient is 2	The seed dispersal distance is between 100 and 1000 m; the value of the coefficient is 2.	Species regulated at the European level: the value of the coefficient for these species is 2.	
	Serious health problem: the value of the health impact coefficient is 3	The seed dispersal distance is greater than 1000 m; the value of the coefficient is 3	Species regulated at the national level: the value of the coefficient for these species is 3.	
				
Acer negundo	1	1	1	1
Ailanthus altissima	2	1	1	1
Ambrosia artemisiifolia	3	3	3	3
Baccharis halimifolia	1	3	2	2
Buddleja davidii	1	3	1	2
Carpobrotus edulis	1	2	1	1
Cortaderia selloana	1	3	1	2
Heracleum mantegazzianum	3	3	2	2
Impatiens glandulifera	1	1	2	1
Phytolacca americana	2	2	1	2
Reynoutria japonica	1	1	1	1
Reynoutria sachalinensis	1	1	1	1
Rhus typhina	2	1	1	1
Robinia pseudoacacia	1	2	1	1
Senecio inaequidens	1	3	1	2
Solidago canadensis	1	3	1	2
Solidago gigantea	1	3	1	2
Ludwigia grandiflora	1	3	2	2
Ludwigia peploides	1	3	2	2

a The distance of seed dispersal: The seed dispersal distance indicates the proliferative capacity of the species. The seed dispersal coefficient can be distributed according to three categories:

b Regulatory status Coefficient: Three categories were determined in according with the list of species listed under the Invasive Plants Regulation in France and the European level:

## 2 Materials and methods

The indicator of the risk of propagation of invasive alien plant species on work zones (IRPW) is the combination of three primary factors

IRPW=FOM*ECF*Cof.sp

### 2.1 FOM: The frequency and abundance of invasive species in the work zone: the factor index of observation at multiple scales

$$\mathrm{FOM}=\frac{\mathrm{average\backslash Frequency\backslash of\backslash Observation}\;\mathrm{of\backslash the\backslash species\backslash inside}\;a\;10\mathrm{km}*10\mathrm{km\backslash area}}{\mathrm{Frequency\backslash of\backslash Observation\backslash of}\;\mathrm{the\backslash species\backslash in\backslash the}\;\mathrm{town}}$$

If the ratio between the frequency of observation in the 10 km*10 km area and the frequency of observation in the town is **less than or equal to 1**, this indicates that the presence of the species in the town where the work zone is higher than the presence of the species in the 10 km*10 km area around the town. The FMO index in this case is 1, the maximum value

If the ratio between the frequency of observation in the 10 km*10 km area and the frequency of observation in the town is **more than 1**, this indicates that the presence of the species in the town where the work zone is **lower** than the presence of the species in the 10 km*10 km area around the town. The FMO index in this case is 0.0001, the minimum value.

### 2.2 ECF The index of environmental conditions that are favorable to the establishment of IEPS

The ECF depends on two factors:

• The type of habitat in which the work zone is located and whether or not it is favorable to the establishment of the species (as indicated by the favorable habitat index
• The proximity of the work zone to a highway, railway, or river, elements that are favoring the proliferation of invasive species

This index can take one of the following values:

A maximum **value of 2** when the habitat is favorable for the species and the work zone is located at less than 200 meters from a highway, railway, or river.

A **value between 1 and 2** when the habitat is favorable for the species and the work zone is located at less than 1 km from a highway, railway, or river.

A **value between 0.1 and 1** when the habitat is not favorable for the species and the work zone is located at less than 1 km from a highway, railway, or river.

A **value of 0.01** when the habitat is not favorable for the species and the work zone is isolated from any dispersal device .

#### 2.2.1 Favorable habitats for species

The favorable habitat for a species describes the characteristics of the environment in which a population of individual members of a given species can live and flourish.

The “Favorable habitat” index is calculated for each work zone for the species present in the administrative district where the work zone is located, and **varies between 1 and 2**.

The **value is 1** when the habitat in which the work zone is located is **not favorable** for the species in question, and is **2** when the habitat is **favorable** for the species in question.

To determine the “Favorable habitat” CORINE Land Cover 2012 [1] database describing land use in France was used more details will follow" on III.3

#### 2.2.2 The proximity Index

Transportation routes, such as roads, railways, and rivers, are a significant anthropic factor that are known to favor the propagation of (IAPS) 7., 13., 16.. In this context, the proximity index measures the proximity of the work zone to highways, railways, and rivers.

The value of this index is 0 when the work zone is at less than 200 meters from a highway, railway, or river. This value increases progressively by 0.1 for every 100 meters of distance, and reaches a value of 0.99 at 900 meters’ distance between the center of the work zone and one of these devices.

#### 2.2.3 Specific coefficient for each invasive species

Invasive exotic plant species are considered a serious threat to plant diversity 8., 10., 18., 14., each invasive species has a particular capacity for proliferation, and a legal status, and they affect human health to a particular degree. The specific coefficient for each invasive species weights the IRPW index as a function of the specific weight attributed to each invasive species.

The specific coefficient for each species depends on three factors: (1) The impact on human health of the species, (2) The distance of seed dissemination and (3) regulatory status of the species (Table 2)

$$\mathrm{Specific\backslash coefficient}=\frac{\mathrm{Health\backslash impact\backslash coefficient\backslash +\backslash Seed\backslash dissemination\backslash coeff\backslash icient}\;\mathrm{+\backslash Regulatory\backslash status\backslash coefficient}}{3}$$
