# Supplementary material for: Data maps and method for evaluating the indicator of the risk of propagation of invasive exotic plant species on work zones
Source: Data Brief. 2018 Jun 26;19:1779–84. doi: 10.1016/j.dib.2018.06.038 (PMC6141259; doi:10.1016/j.dib.2018.06.038)
Supplement: Supplementary file 1 — Supplementary material [file mmc5.pdf]

Conflicts of Interest Statement

Manuscript title: ____

____

____

The authors whose names are listed immediately below certify that they have NO affiliations with or involvement in any organization or entity with any financial interest (such as honoraria; educational grants; participation in speakers' bureaus; membership, employment, consultancies, stock ownership, or other equity interest; and expert testimony or patent-licensing arrangements), or non-financial interest (such as personal or professional relationships, affiliations, knowledge or beliefs) in the subject matter or materials discussed in this manuscript.

Author names:

The authors whose names are listed immediately below report the following details of affiliation or involvement in an organization or entity with a financial or non-financial interest in the subject matter or materials discussed in this manuscript. Please specify the nature of the conflict on a separate sheet of paper if the space below is inadequate.

Author names:

This statement is signed by all the authors to indicate agreement that the above information is true and correct (*a photocopy of this form may be used if there are more than 10 authors*):

Author's name (typed)	Author's signature	Date
